# The influence of spaceflight and simulated microgravity on bacterial motility and chemotaxis

**DOI:** 10.1038/s41526-021-00135-x

**Published:** 2021-02-22

**Authors:** Jacqueline M. Acres, Myka Jaap Youngapelian, Jay Nadeau

**Affiliations:** Portland State University, Portland, OR USA

**Keywords:** Biophysics, Microbiology, Fluid dynamics

## Abstract

As interest in space exploration rises, there is a growing need to quantify the impact of microgravity on the growth, survival, and adaptation of microorganisms, including those responsible for astronaut illness. Motility is a key microbial behavior that plays important roles in nutrient assimilation, tissue localization and invasion, pathogenicity, biofilm formation, and ultimately survival. Very few studies have specifically looked at the effects of microgravity on the phenotypes of microbial motility. However, genomic and transcriptomic studies give a broad general picture of overall gene expression that can be used to predict motility phenotypes based upon selected genes, such as those responsible for flagellar synthesis and function and/or taxis. In this review, we focus on specific strains of Gram-negative bacteria that have been the most studied in this context. We begin with a discussion of Earth-based microgravity simulation systems and how they may affect the genes and phenotypes of interest. We then summarize results from both Earth- and space-based systems showing effects of microgravity on motility-related genes and phenotypes.

## Background

Earth-based organisms, including microorganisms, have developed under the influence of gravity. As interest in spaceflight grows, it is important to understand the effects of microgravity on single-celled organisms including Bacteria, Archaea, and eukaryotes such as yeast and microalgae. In the microgravity environment particles experience weightlessness, such as in the case of constant free-fall in orbit aboard the International Space Station (ISS). The effects of microgravity exposure on microorganisms are of intense interest for medical and bioengineering applications, and many studies have been conducted over the past few decades, of which there are several comprehensive reviews^1–6^. However, a systematic summary of the effects of microgravity on microbial motility has not yet been done. Microbial motility is important for normal function of the human microbiome (e.g., in the gut and oral cavity)^7,8^ as well as for pathogenesis of some bacteria involved in common food- and water-borne infections^9–12^. Alterations in motility affect the distribution of microorganisms in tissues, encourage or inhibit bacterial invasiveness, and affect biofilm formation^11,13^. These changes may have important implications for astronaut health, especially combined with host factors; studies suggest that astronauts aboard the ISS suffer from compromised immune systems^14,15^ and altered microbiota^16^, potentially making them susceptible to opportunistic bacterial infections.

Microorganisms rapidly adapt to their environment by altering their gene expression to increase survivability. While earlier studies of microgravity effects on microorganisms were largely phenotypic, more recently “omics” techniques have become practical: genomics, transcriptomics, and proteomics, quantifying DNA, mRNA, and proteins, respectively^17–19^. Genomic studies are the most prevalent and in general the easiest to perform for bacteria, since many organisms have been fully sequenced and DNA-sequencing technology is widely available and relatively inexpensive^20^. RNA sequencing remains costly for transcriptomic studies, although less expensive microarray technologies may also be used^19^. Proteomics is a rapidly emerging field, but fractionation of bacteria for proteomic studies, particularly Gram-negative strains, is challenging^21,22^. Beginning with microarray studies of simulated microgravity responses in 2002^23^, gene expression in microbes has been studied in both ground-based^24–26^ and spaceflight studies^27,28^. Transcriptomic and proteomic studies have also begun to appear^29,30^. Development of technologies for “omics” studies in space has been progressing but is not yet routinely used^31^. Sorting through the plethora of information available is rapidly becoming increasingly difficult, with numerous databases and software tools devoted to the task^32–35^.

An important consideration is the relationship between the presence of motility genes (and their transcripts) and phenotype. The genes of the flagellar regulon are expressed as a cascade, and transcriptional activators influence expression of motility genes based upon environmental conditions^36,37^. This illustrates the importance of performing linked transcriptomic/proteomic and phenotypic studies when working with a test strain to study changes over short time periods (minutes to hours). Despite this complexity, the overall presence of flagellar and chemoreceptor genes has been used to predict community-wide motility behaviors^38,39^; combined “omics” studies (genomic/transcriptomic) can capture diel and seasonal variations in motility^40^.

**Fig. 1:**
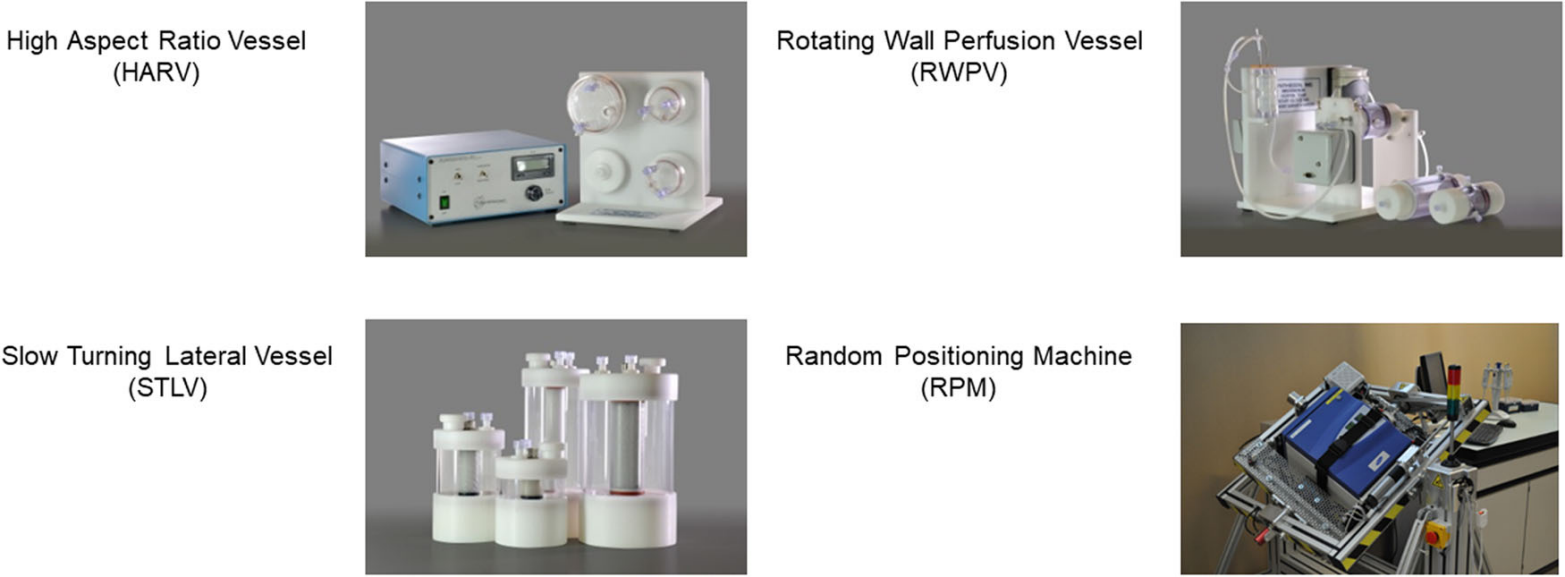
Ground-based systems for simulating microgravity. HARV, STLV, and RWPV images from Synthecon, Houston, TX with permission from Bill Anderson. RPM image reprinted from Wuest et al.^139^ with no modification under the CC BY 3.0 License (https://creativecommons.org/licenses/by/3.0/).

Spaceflight analog devices used on Earth, such as those shown in Fig. 1, mimic specific hallmarks of the microgravity environment including low fluid shear, lack of sedimentation, and low turbulence^41^. Thus, we will refer to the culture environment in spaceflight analog devices as ‘simulated microgravity.’ The impact of simulated microgravity studies goes beyond prediction of microbial changes during spaceflight. In fact, low fluid shear conditions also exist in vivo under special circumstances, such as in the microvilli of the intestines^42^. In these environments, organisms colonize and infect their hosts. Therefore, simulated microgravity devices can also be an invaluable tool to understanding mechanisms of the infection process^43–45^.

This review begins by summarizing the technologies available for simulating microgravity, with some recent analysis of the fluid mechanics of spaceflight analog devices. The possible effects of the different types of vessels on microorganisms apart from their simulation of weightlessness are discussed, as well as other complications that may arise from attempting to compare ground-based with ISS-based microbial gene expression studies. We then detail recent studies in gene expression regarding motility and chemotaxis in selected Gram-negative bacterial strains: *Escherichia coli*, *Salmonella enterica* serovar Typhimurium, *Pseudomonas aeruginosa*, and *Vibrio fischeri*.

## Simulated microgravity and fluid mechanics

Due to the difficulty of conducting experiments in space, ground-based bioreactors were developed by the NASA Johnson Space Center (Houston, TX) to study simulated microgravity on Earth^46^. These devices are generally called rotating wall vessels (RWV), though this terminology can be confusing^2,47^ as it includes different configurations such as: high aspect ratio vessels (HARVs)^48^, slow turning lateral vessels (STLVs)^46,49^, and rotating wall perfused vessels (RWPVs)^50^. Other devices used to simulate microgravity and of slightly different design are clinostats^51^ and random positioning machines (RPMs)^52^. Though they have been used to study simulated microgravity on plants^51^, and human cells and tissues^53–55^, this review’s focus is limited to effects on microbes.

Fluid behavior under simulated microgravity is characterized by low fluid shear, often referred to as low shear modeled microgravity (LSMMG)^23^. Low fluid shear values associated with the simulated microgravity environment are of the order ~10^–2^ dyne/cm^2^^43,50^. The low fluid shear condition is dependent on many factors including vessel geometry, particle size, vessel rotation speed, and fluid properties. Though all vessel types create simulated microgravity, analysis differs slightly among types. Flow characterization can be approximated with the Reynolds number (Re), which is a ratio of the inertial to viscous forces. For example, for flow in a cylindrical vessel, the Reynolds number is given by Eq. (1):

1 $${\mathrm {Re}} = \frac{{\rho VD}}{\mu }$$

where *ρ* is the fluid density, *V* is the fluid velocity, *D* is the particle diameter, and *μ* is the kinematic viscosity.

**Fig. 2:**
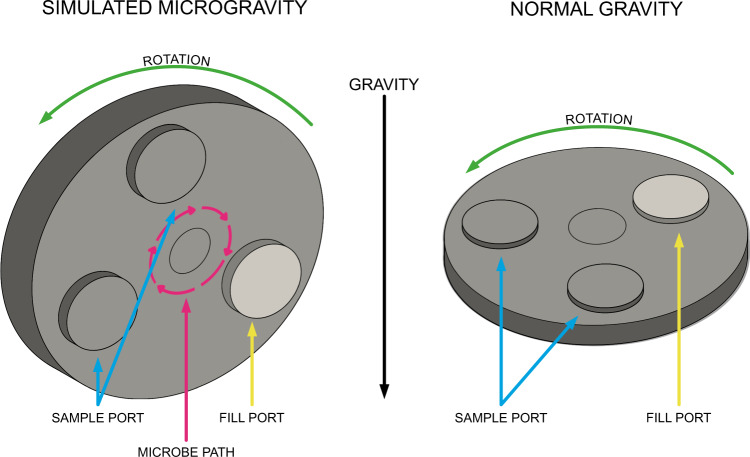
3D orientation of a rotating wall vessel (RWV) with the direction of the gravity vector included. Left: Simulated microgravity showing a typical microbe path with gravity vectors canceling in the completely vertical orientation. Right: Normal gravity control with a horizontal orientation.

Common principles and the development of RWVs have been outlined in many sources^41,50,56^. Ground-based systems replicate weightlessness by rotating cells such that gravity vectors are nullified, and organisms do not have the opportunity to adapt to a specific gravity orientation. In other words, gravity is not altered; rather, the summation of gravity effects cancels out^41,57^. When the vessel is filled completely (zero headspace), the chamber contents resemble a rigid body. Fluid within the vessel is assumed both incompressible and Newtonian with approximately uniform density and viscosity. Vessel orientation determines whether microbes are under normal gravity (vertical axis) or simulated microgravity (horizontal axis) as seen in Fig. 2. A common method of control is to inoculate two chambers and grow cells in both configurations. However, caution should be exercised when comparing differences between these two configurations. It is possible that there are fluid dynamic effects under simulated microgravity that do not appear in the normal gravity configuration^58,59^. Different studies have employed both analytical and numerical techniques such as computational fluid dynamics (CFD) to elucidate the fluid mechanical effects and when they must be considered. This review aims to provide a brief overview of these analyses specific to device type when possible, focusing on those devices primarily used in microbial-simulated microgravity studies.

We begin with HARVs^48^, as these devices were used for most simulated microgravity studies presented in this review. These devices are cylindrical in shape with a membrane on the back to facilitate gas exchange^60^. Ayyaswamy and Mukundakrishnan^59^ outlined experimental conditions necessary for simulated microgravity in HARVs and STLVs including low fluid shear at the cell surface, adequate mass transport, and if microcarriers are used, such as when studying biofilm development^61^, to exercise careful thought about size and shape so as to minimize collisions with the vessel walls and adverse fluid effects.

The STLV^49^ consists of an inner and an outer cylinder, both of which may rotate along a horizontal axis, in either the same or opposite directions. Oxygenation is provided through a membrane along the inner column. The fluid behavior was initially reviewed in Hammond and Hammond^41^ in 2001, and further studies have been done since then. Gao et al.^62^ showed that particles tended to migrate radially when the particle density and fluid density differed. Liu et al.^63^ later replicated and expanded on this work, providing a comprehensive analysis of the forces on the particle. They also proposed rotational speeds consistent with simulated microgravity on inert particles and reaffirmed difficulties present with larger particle sizes, reporting fluid shear stress values several orders of magnitude higher than simulated microgravity.

**Fig. 3:**
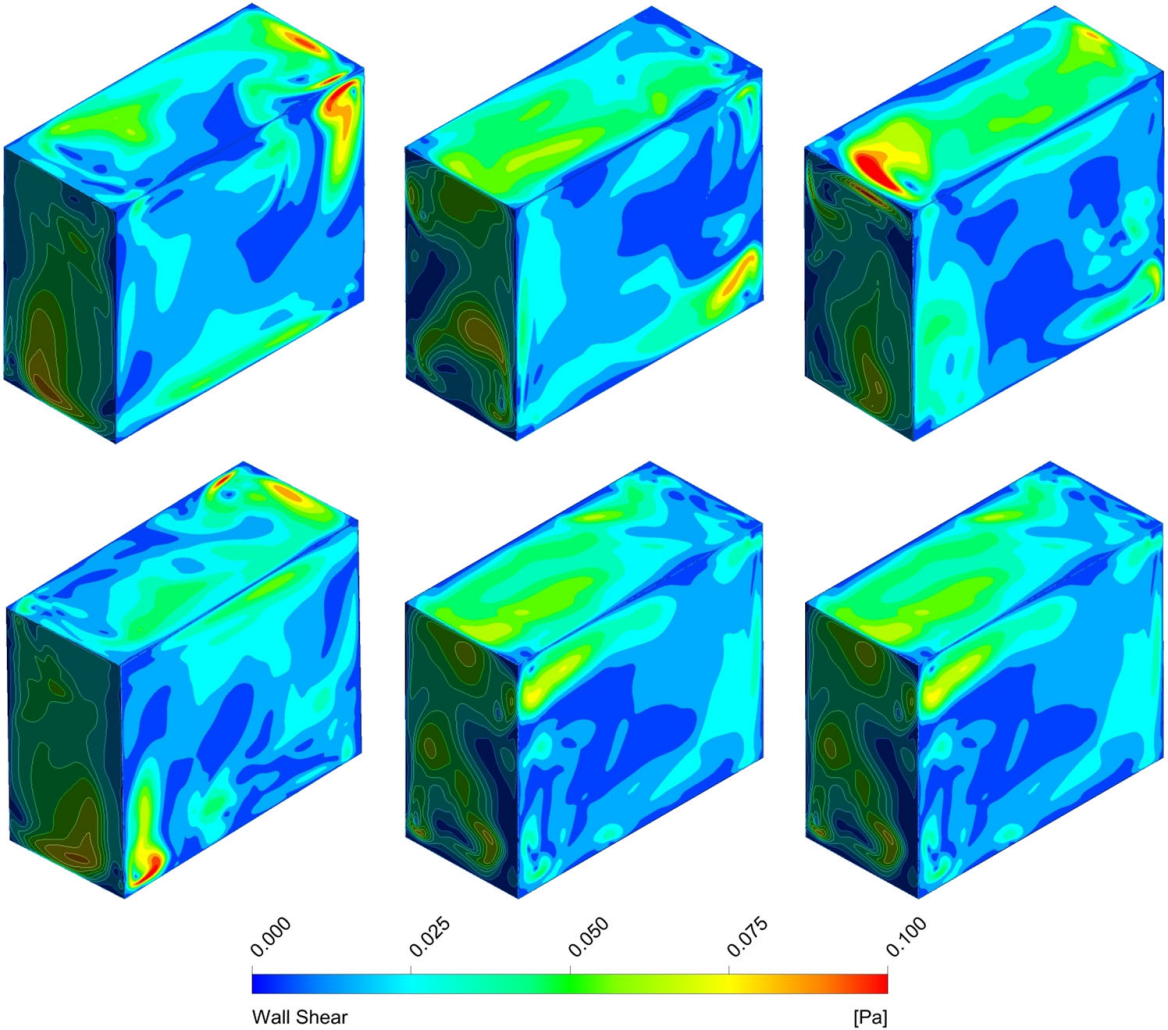
Simulated shear stress values along the walls in an RPM. Shear stress values vary throughout with no clear steady state. Reprinted from Wuest et al.^58^ with no alterations under the CC BY 4.0 License (https://creativecommons.org/licenses/by/4.0/).

The RPM was developed from the clinostat^52^, and consists of two frames that can be independently driven. Therefore, an RPM can be operated in different modes^64^: clinostat mode (only one axis rotates), 3D clinostat mode (two axes rotated at constant speed), and 3D Random mode (two axes rotated at different speeds). Wuest et al.^58^ studied the fluid motion using a numerical approach, comparing the classic clinostat rotation with the 3D clinostat mode. They found that the fluid motion in the cell flask varied as a function of position and never seemed to reach a steady state, as seen in Fig. 3. Fluid shear stress values were found to be highest at the walls and could reach up to a 100 mPa. They urge that experimental design on microbes or tissues must consider fluid dynamic effects.

**Fig. 4:**
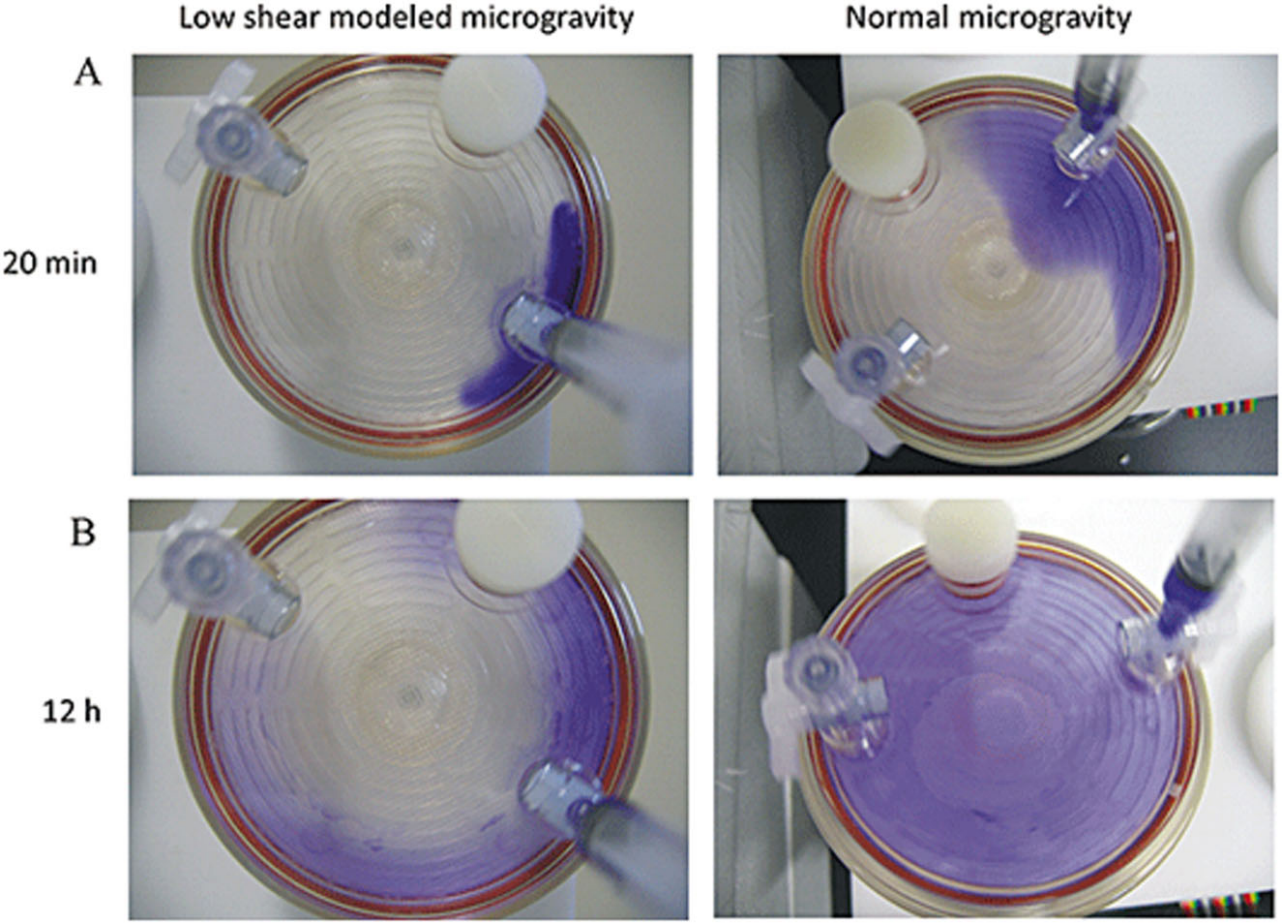
HARV bioreactors injected with crystal violet. (Left) Under simulated microgravity, the dye stays along the outer wall before gradually migrating inward. (Right) The dye spread under normal gravity. Reprinted from Crabbe et al. ^65^ with permission from John Wiley and Sons with no alterations.

Although each device simulates microgravity, they do not all do so in the exact same manner. Consequently, organisms grown in one device may not react the same as another device^47^. When conducting a study of *P. aeruginosa*, Crabbe et al.^65^ found differing levels of gene expression when culturing in a HARV vs. in an RPM. They probed this difference by injecting dye into one HARV port as seen in Fig. 4. In their supplemental videos, they show how the dye spread to the center of chamber more slowly in the HARV than in the RPM indicating a subtle difference in fluid behavior. This could be a possible reason for the difference in gene expression.

### Simulated microgravity and imaging

Experimental calculation of fluid shear stress requires estimation of particle velocities^41,50,66^, but imaging under simulated microgravity conditions on Earth is challenging. Although any of the traditional methods described above can be used to study simulated microgravity, changes in gene expression can occur in minutes. This is particularly relevant for observing dynamic phenotypic changes in motility and chemotaxis. To address this, one approach is to attach a microscope to a simulated microgravity device, as was done with the clinostat microscope^67^ developed by the German Aerospace Research Establishment in 1996. The microscope is positioned to rotate horizontally and was used to study the behavior of *Paramecium biaurelia* in both simulated microgravity and spaceflight^68^. Another example of this was when Pache et al.^69^ attached a digital holographic microscope to an RPM, upgraded by Toy et al.^70,71^ to include a widefield epifluorescence microscopy module. Yew et al.^72^ developed a lab-on-a-chip clinorotation system as a more cost-effective alternative to the systems previously described. It uses 2D clinostat rotation and requires pauses for imaging.

## Simulated microgravity, spaceflight and gene expression

**Fig. 5:**
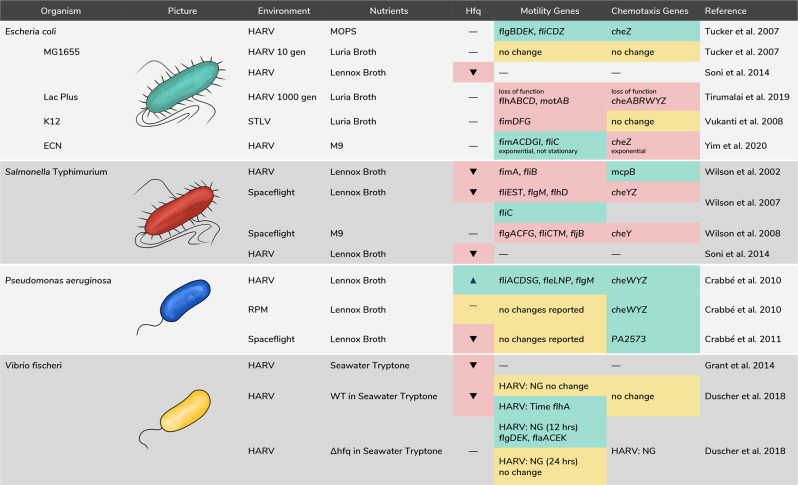
Motility and chemotaxis gene expression categorized by study and organism. Color indicators: Green: upregulation, Yellow: no change reported, Red: downregulation. Dashes indicate no data available. Normal Gravity Rotation control is represented by NG.

Gene regulation is a process in which gene expression is upregulated or downregulated and can be influenced by environmental factors. Here we report the influence of simulated microgravity and spaceflight on gene expression involving *hfq* regulation^23,27,29,65,73–75^, motility-related systems (flagella or fimbriae)^23,26,27,29,30,65,74,76–79^, and chemotaxis-related systems (chemical sensing fimbriae, pH sensing, media sensing)^23,26,27,29,30,65,74,76–79^. Results are illustrated in Fig. 5 with more details to follow. First, we provide a brief overview of *hfq*, motility and chemotaxis followed by organism-specific spaceflight and simulated microgravity studies.

### Overview of *hfq*, motility and chemotaxis *hfq*

#### *hfq*

Hfq has emerged as an important post-transcriptional factor that facilitates the pairing of small RNAs with their target mRNAs; its role in bacteria has been recently reviewed^80–82^. To highlight its importance, in some organisms it can impact expression of up to 20% of all genes^83^. In a landmark study in 2007, Wilson et al.^27^ first identified Hfq as a global regulator in response to the spaceflight and simulated microgravity environment based on their global microarray and proteomic analyses. Additionally, changes in *hfq* gene expression can influence virulence of bacterial pathogens^83^ including *S*. Typhimurium^83,84^ thus making this gene a recent focus in simulated microgravity studies^27,29,30,44^.

#### Motility and chemotaxis

**Fig. 6:**
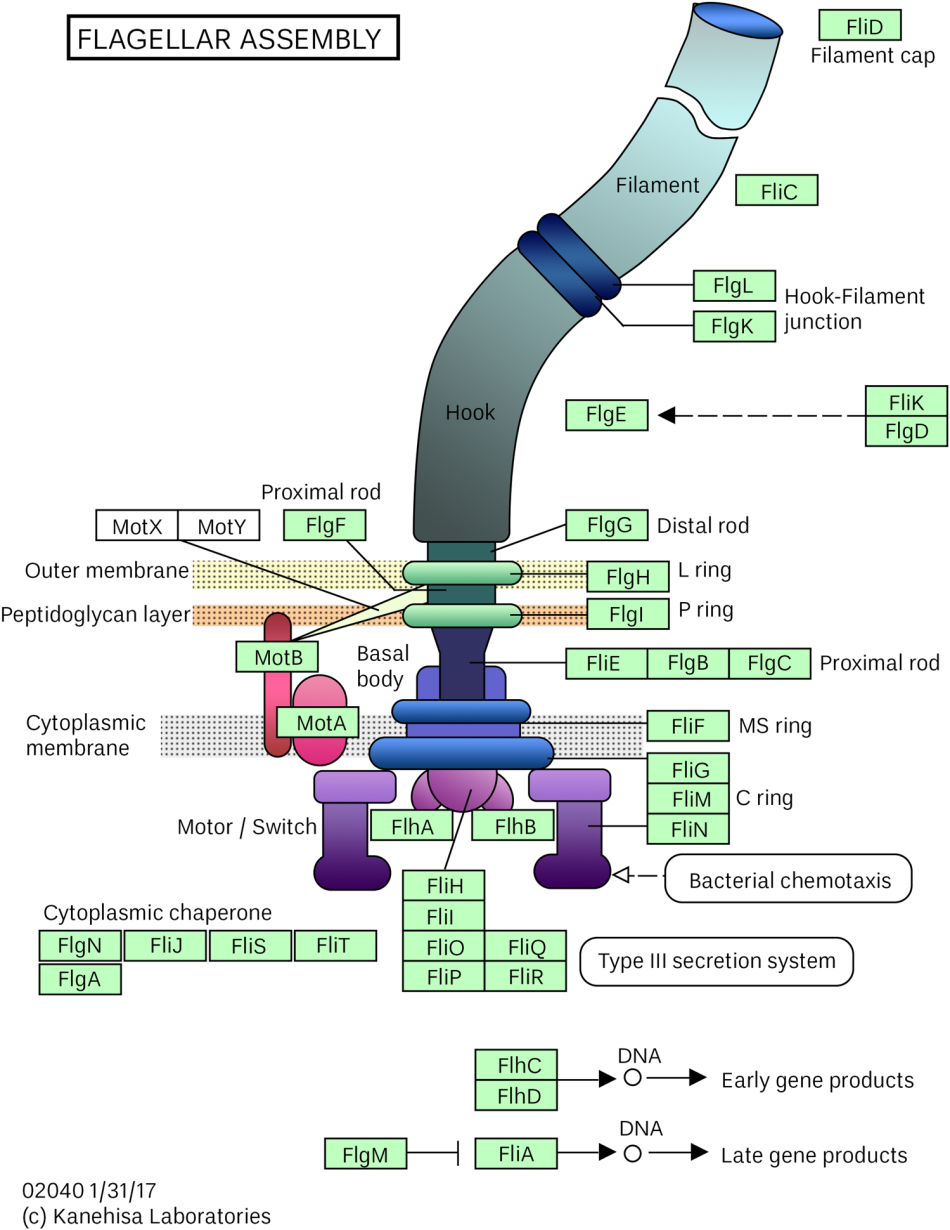
Bacterial flagellar motor (*E. coli*) with associated protein components. Eco02040 reprinted with modification under the CC BY 4.0 License (https://creativecommons.org/licenses/by/4.0/) based on KEGG, Kanehisa et al.^88^.

Changes in microbial motility can result from alterations in gene expression of motility machinery including flagella, fimbriae, and pili. Motor assembly and chemosensory machinery requires about 50 genes in *E. coli* and *S*. Typhimurium^85^. Studies have been conducted to identify genes^86^ and proteins^87^ involved in bacterial motility, with the *E. coli* flagellar assembly shown in Fig. 6^88^. Flagellar genes are organized into regulatory hierarchies of classes depending on the organism, with each hierarchy affecting the next^37,89,90^. Flagella are not only important for motility^85^; they have also been shown to play a role in pathogenicity^9,91–93^ and biofilm formation^13,94^.

**Fig. 7:**
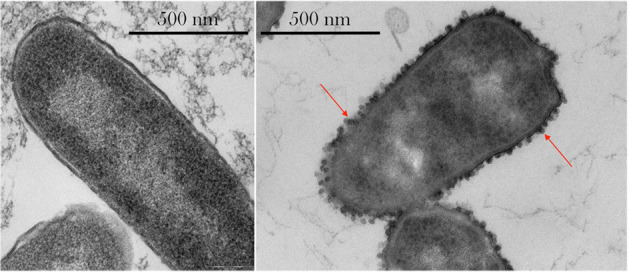
Thin-section transmission electron microscopy images of *E. coli*. Left: Sample cultures on Earth. Right: Sample cultured in space exhibiting an irregular cell shape. Red arrows indicate extracellular vesicles. Images taken with a Phillips CM 100 TEM at an accelerating voltage of 80 kV. Figure and modified caption reprinted from Zea et al.^96^ under the CC BY 4.0 License (https://creativecommons.org/licenses/by/4.0/).

Motility and cell shape have been shown to influence cell growth^95^. Interestingly, transmission electron microscope (TEM) images have shown phenotypic changes in *E. coli* after spaceflight, with decreased size and increased presence of outer membrane vesicles as seen in Fig. 7^96^. Benoit and Klaus^3^ reviewed microbial motility as it related to final cell populations, comparing motile and non-motile cultures during spaceflight, simulated microgravity and normal gravity controls. They revealed that spaceflight and simulated microgravity compared to normal gravity controls resulted in higher final cell counts in non-motile bacteria grown in liquid. Microbial motility is governed by an electromotive gradient of ions across the cell membrane, so concentration differences or nutrient depletion due to microgravity or simulated microgravity can affect cell motility^85^.

Chemotaxis is the response of a cell to a chemical gradient. Adler’s pioneering work^97^ in *E. coli* chemotaxis showed that movement towards attractants is independent of the mechanistic benefit of the compound itself. Although taxis components can vary between species, there are core components to all prokaryotic chemotactic systems^98,99^. Genomic analysis of chemotaxis shows that methyl-accepting chemotaxis proteins (MCPs), CheA and CheW are present in >95% of prokaryotic genomes that contain at least one chemotaxis gene, and CheBYR are common in ~90% of prokaryotic genomes^100^.

### *E. coli*: *hfq*, motility, and chemotaxis

*E. coli* is an organism with motile and non-motile strains and is the most well studied and most thoroughly characterized organism on Earth^101^. *E. coli* K12 MG 1655 is motile with peritrichous flagella and fully sequenced^102^. *E. coli* Nissle (EcN) 1917 is another fully sequenced^103^, probiotic, non-pathogenic strain of *E. coli* lacking pathogenic adhesion factors; it carries genes that help limit the proliferation of other bacteria^104^. *E. coli* K12 MG 1655 has shown increased biofilm formation under simulated microgravity^61^.

#### *E*. *coli* and hfq

Tsui et al.^105^ showed that an *hfq* insertion mutation caused pleiotropic phenotypes, thus highlighting the importance of *hfq* expression in *E. coli*. Soni et al.^73^ studied the response of *hfq* and *trp* genes under simulated microgravity for various enterobacteria. Cultures of *E. coli* MG1655, DH5α, and AS11 were grown in Lennox broth using HARVs and compared with normal gravity rotation. They found *hfq* expression to be downregulated.

#### *E. coli*: motility and chemotaxis

In *E. coli*, motility is critical for biofilm formation^94^. Tucker et al.^76^ cultured *E. coli* MG1655 in both minimal MOPS medium and Luria broth, comparing cultures in HARVs vs. normal gravity rotation. In the MOPS media, flagellar genes *flgBDEK* and *fliCDZ* were upregulated, whereas no changes were observed with the Luria broth compared to normal gravity controls, respectively. Regarding chemotaxis, they found an upregulation of *cheZ* in the MOPS media vs. normal gravity rotation. Motility and chemotaxis are used by bacteria to identify and obtain nutrients; thus, expression levels vary with the level of nutrients in the environment. This study underlines the importance of this factor in designing studies of motility changes under simulated microgravity.

Tirumalai et al. conducted two studies^77,79^ analyzing gene mutation following different HARV cleaning protocols. In one study^79^, the HARVs were cleaned using steam sterilization; in a later study^77^, they used chloroamphenicol to prevent contamination. In both studies, they cultured 1000 generations of *lac*+ and *lac*− cultures of *E. coli* 1655, chosen because they could be visually distinguished. Following the chloramphenicol treatment, the *lac*+ cultures showed mutations in the predicted fimbriae-like adhesion proteins *yadL*. Flagellar and motility proteins also had loss of function mutations *flhABCD*, *motAB*. Since these changes were not present in the steam sterilization study, they were thought to aid in antibiotic resistance under simulated microgravity conditions. Additionally, they found loss of function mutations in the chemotaxis-related genes *cheABRWYZ*. Although they did not look at gene expression changes in motility specifically, Lynch et al.^61^ found that biofilm coverage of *E. coli* on microcarrier particles cultured in Luria Bertani broth in 10 mL HARVs was more pronounced than under normal gravity. Vukanti et al.^26^ cultured *E. coli* in Luria broth in an STLV and found a downregulation of *fimDFG*. Yim et al.^106^ grew EcN in M9 minimal media in 10 mL HARVs. Sample collection followed exponential and stationary growth. They found upregulation of *fliC*, *fimACDGI* after exponential growth, but changes did not persist in samples taken from stationary growth. Regarding chemotaxis, they also found a downregulation of *cheZ* following the same conditions.

### *S*. Typhimurium: *hfq*, motility and chemotaxis

*S*. Typhimurium is motile with peritrichous flagella, a fully sequenced^107^ pathogenic organism shown to have increased virulence after simulated microgravity exposure^108^ and spaceflight^27,74^.

#### *S*. Typhimurium and *hfq*

In non-space-related studies, Sittka et al.^109^ showed that in *S*. Typhimurium Hfq influences 87% of genes in the flagellar system and 84% of genes in the chemotaxis system. Monteiro et al.^110^ showed that Hfq controls biofilm formation through regulation of CsgD in *S*. Typhimurium. Since changes in *hfq* expression have been shown to impact expression of genes related to motility^109^, chemotaxis^109^, and biofilm formation^110^ in *S*. Typhimurium in Earth-based studies, studies were conducted to ascertain whether these changes persisted under simulated microgravity and spaceflight. In a study conducted by Wilson et al. of bacterial gene expression after spaceflight^27^, *hfq* expression was down-regulated. Noting that simulated microgravity induced acid resistance in a previous study at late log phase^23^, they cultured both the wild type and an *hfq*-deficient mutant in Lennox broth in HARVs, comparing survivability. The wild type showed greater survivability in normal gravity rotation vs. simulated microgravity. However, this difference in survivability was not observed between simulated microgravity and normal gravity rotation for the *hfq*-deficient strain. Pacello et al.^111^ cultured *S*. Typhimurium in Luria Bertani broth in 10 mL HARVs using normal gravity as a control. They found that simulated microgravity increased acid resistance even in the absence of *hfq* as compared to normal gravity. Soni et al.^73^ cultured *S*. Typhimurium in Lennox broth in HARVs using normal gravity rotation as a control. They observed a downregulation of *hfq*.

### *S*. Typhimurium: motility and chemotaxis

*S*. Typhimurium motility has been shown to increase invasiveness^9^, therefore making changes in motility important from a health perspective. Wilson et al.^23^ compared cultures of *S*. Typhimurium grown in Lennox broth in HARVs and normal gravity, finding 163 genes differentially expressed under simulated microgravity, including downregulation of *fimA, fliB*. They reported an upregulation of *mcpB*, a gene identified in chemotaxis in *S*. Typhimurium^112^. In a later study^27^, they compared spaceflight *S*. Typhimurium samples with controls on the ground kept under similar temperature and nutrient conditions. They showed downregulation in *fliEST, flgM, flhD*; however, *fliC* was upregulated. Regarding chemotaxis, they found a downregulation of *cheYZ*. In another study^74^, they cultured *S*. Typhimurium in M9 media during spaceflight and showed eight genes in *flgACFG, fliCMT, fljB* downregulated compared to ground-based controls. Additionally, they found a downregulation of *cheY*.

### *P*. *aeruginosa*: *hfq*, motility and chemotaxis

*P. aeruginosa* is motile with a single polar flagellum, fully sequenced^113^, and occasionally a part of human flora. In spaceflight^114^ and simulated microgravity^29^, it has shown increased biofilm formation.

#### *P. aeruginosa* and hfq

Crabbe et al.^65^ investigated global changes in gene expression including the *hfq*-dependent response under simulated microgravity using both a HARV and an RPM in *P. aeruginosa* PAO1. *Hfq* was upregulated using the HARV as compared to normal gravity rotation, but not differentially expressed when comparing the RPM to normal gravity rotation. In *P. aeruginosa*, the loss of *hfq* can result in reduction in growth^115^. Interestingly, comparison of total bacterial counts showed no significant differences between HARV, RPM, and normal gravity. Crabbe et al.^29^ also investigated the *hfq*-dependent response after spaceflight, comparing their results with their earlier simulated microgravity study. In contrast to the HARV results, they found a downregulation of *hfq* after spaceflight. This finding was significant because following *S*. Typhimurium, it showed Hfq as a regulator acting across bacterial species.

#### *P. aeruginosa*: motility and chemotaxis

*P. aeruginosa* is associated with infections in immunocompromised hosts. Motility, particularly the presence of *fliC*, plays a key role in its pathogenesis^91^. Crabbe et al.^65^ cultured *P. aeruginosa* in Lennox broth in HARVS and RPMs and cataloged changes in motility, finding upregulation of motility genes *fliACDGS*, *fleLNP, flgM* but no apparent changes under the RPM. No significant changes in motility gene expression were reported after spaceflight. In contrast to what is seen in *E. coli* and *S*. Typhimurium, the chemotactic response pathway in *P. aeruginosa* involves more than 20 *che* genes and 26 MCP-like genes^116^. Crabbe et al.^65^ showed upregulation of chemotaxis genes *cheWYZ* in the HARV and to a lesser extent in the RPM compared to normal gravity. Spaceflight^29^ showed an upregulation of *PA2573*, an MCP homolog^117^ compared to ground controls. Kim et al.^114,118^ compared biofilm formation in *P. aeruginosa* PA14 during spaceflight with ground controls finding that spaceflight not only increased biofilm formation but also revealed a different biofilm architecture than normally appears on Earth that they termed “column-and-canopy”. Additionally, they investigated biofilm formation in the wild type, mutants deficient in flagella-driven motility, Δ*motABCD* and type IV pili-driven motility Δ*pilB*. The wild type and Δ*pilB* made the column-and-canopy architecture, while the Δ*motABCD* did not, thus underscoring the importance of motility in the formation of this architecture.

### *V. fischeri*: *hfq*, motility and chemotaxis

*V. fischeri* is a motile marine bacterium with a single polar flagellum, completely sequenced^119^. It forms a symbiotic relationship with the bobtail squid *Euprymna scolopes* and as such is a good model for understanding how such relationships can change in simulated microgravity^120^.

#### V. fischeri and hfq

*Hfq* expression was also studied in *V. fischeri* and its resulting effect on *E. scolopes*. Grant et al.^75^ placed both symbiotic partners in HARVs and examined changes in *V. fischeri’s* colonization of *E. scolopes* at different stages of development, using both the wild type and a mutant with a non-functioning *hfq* gene. Under simulated microgravity, both the wild type and mutant reached higher cell counts than under normal gravity. *Hfq* was also downregulated under simulated microgravity. No differences in the ability of *V. fischeri* to colonize *E. scolopes* were observed between the wild type and *hfq* mutant, though colonization occurred more quickly under simulated microgravity with both strains. *V. fischeri* is also critical for morphogenesis of the light organ in *E. scolopes* by triggering developmental events^121^. The wild type, during simulated microgravity, negatively impacted these developmental events, while the *hfq* mutant did not. This coupled with the fact that the *hfq* mutant under normal gravity also negatively impacted these developmental events showed that *hfq* while not necessary for colonization, was still necessary for light organ morphogenesis.

#### *V. fischeri*: motility and chemotaxis

**Fig. 8:**
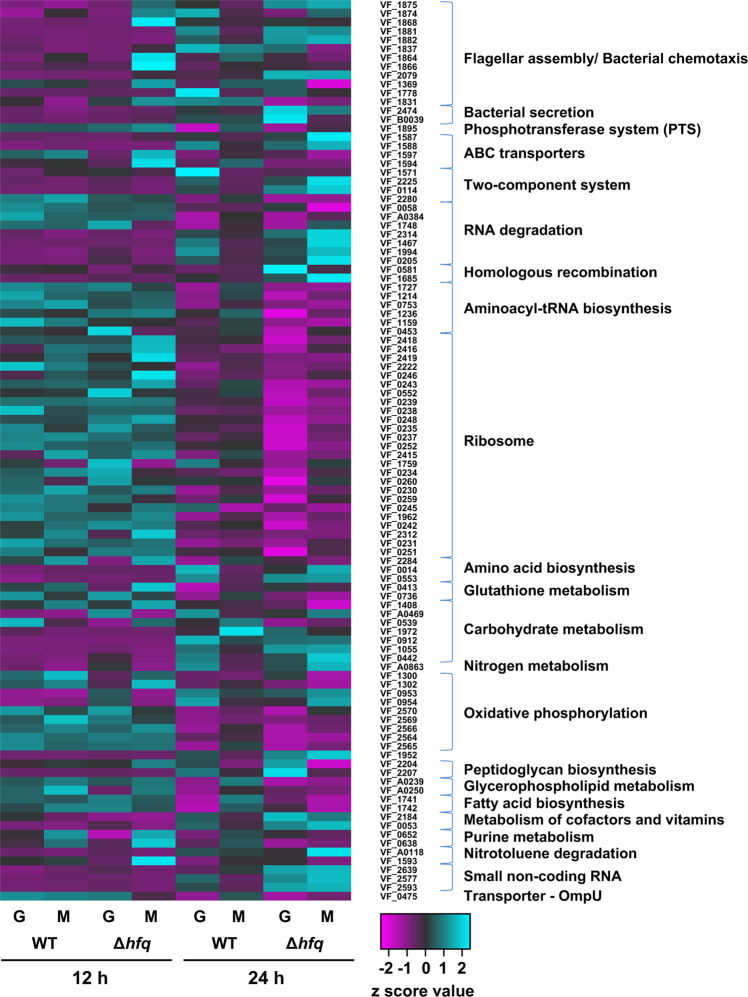
Heat map depicting the clustering patterns of the eight treatments by KEGG pathways associated with the proposed function of *V. fischeri* genes at 12 and 24 h. Gene changes governing flagellar assembly and bacterial chemotaxis labeled at the top. Colors represent the differential abundance of individual genes listed by *V. fischeri* identification number (VF-ID) for both wild type (WT) and Δ*hfq* mutants under simulated microgravity (M) and normal gravity (G) conditions. Figure and modified caption reprinted from Duscher et al.^30^ under the conditions of the CC BY 4.0 License (https://creativecommons.org/licenses/by/4.0/).

Motility is necessary for *V. fischeri* to successfully colonize *E. scolopes*^122^. Duscher et al.^30^ investigated *V. fischeri* exposure to simulated microgravity using HARVs. The 12- and 24-h cultures of *V. fischeri* (wild type) and associated global regulator *hfq* protein knockout strains (∆*hfq*) were grown in seawater tryptone. They used a heat map to qualitatively depict changes in gene expression as shown in Fig. 8^30^. When comparing the wild type grown in simulated microgravity vs. normal gravity after 12 and 24 h, no genes showed significant differences. However, comparison of the wild type after 12- and 24-h exposure to simulated microgravity showed an upregulation in *flhA* similar to an upregulation of *flgEM* when viewing changes under normal gravity. The ∆*hfq* strain after 12 h under simulated microgravity showed upregulation of *flgDEK, flaACEK* as compared to normal gravity. After 24 h under simulated microgravity, these changes did not seem to persist. The ability to chemotax is an advantage for *V. fischeri* when colonizing *E. scolopes*, though not strictly necessary^123^. Duscher et al.^30^ did not report many changes to chemotaxis gene expression. Under simulated microgravity, only the ∆*hfq* showed a downregulation of *V. fischeri* chemotaxis genes.

## Conclusions

There is still much work needed to understand microbial response to microgravity. Gene expression can vary based on environmental factors including temperature, nutrients, and fluid shear stress ranges. A recent study argued that there is no identifiable common bacteria “spaceflight response,”^124^ although another suggested Hfq as a general spaceflight regulon^27^.

The two gravity-dependent processes believed to most influence bacteria are indirect, namely (a) settling (of both cells and nutrients) and (b) buoyant convection^125^. Both affect non-motile microorganisms more than motile cells, since flagellar motility stirs the liquid surrounding the cell as well as permitting the cell to avoid settling. Thus, increased growth seen under microgravity conditions was initially hypothesized to be restricted to non-motile cells^3^; however, later studies showed that this was not universally the case. Instead, phosphate and/or oxygen availability are decreased in microgravity due to lack of convection, leading to altered microbial behavior, such as in *P. aeuginosa*^118^. Reduced convection leading to substrate concentration gradients has been proposed as a general mechanism underlying all microbial alterations seen in microgravity^126^. These models are supported by studies using diamagnetic levitation to simulate weightlessness. Levitation prevents settling but increases convection because of the diamagnetic properties of oxygen^127^.

What is known about low-oxygen and low-phosphate environments can help inform microgravity studies, but careful attention to experimental and strain differences is essential. Low-nutrient environments have different effects on motility depending upon the motility features (e.g., run-and-tumble vs. forward-reverse^128^) and stress responses of the particular strain. A significant number of studies have been done with human pathogens, since limiting environments are frequently encountered during the infection process, for example in intestinal villi, and can trigger the transition from planktonic to biofilm in many Gram-negatives^129^. Reduced phosphate leads to increased virulence and swarming motility in *P. aeruginosa*^130^. Oxygen availability influences microbial pathogenicity at all stages of the infection process^131^; *S*. Typhimurium grown in low oxygen environments shows greater adhesion to and invasion of epithelial cells^132^. In the mutualistic *V. fischeri*, which transitions from motile to non-motile as it enters stationary phase, the low-nutrient microenvironments in microgravity may simulate the transition to stationary phase^30^.

Downregulation of *hfq* expression was the most consistent finding in the studies we focused on here and is a common theme in stress-response studies as well^133–136^. As mentioned previously, Hfq is a global transcriptional regulator that has been found in approximately half of all known bacterial genomes. It is an RNA chaperone which can serve as both a positive and a negative regulator. Hfq stabilizes small RNAs (sRNAs) and acts as a platform for sRNA–mRNA interaction; regulation by sRNAs requires Hfq. Hfq-dependent sRNAs play a key role in regulation of flagellar genes by acting on the master regulator, FlhD, as well as other factors (refer again to Fig. 6)^137^. Almost 90% of flagellar genes are Hfq-regulated in common Gram-negative pathogens^138^. The general downregulation of *hfq* expression seen in spaceflight and simulated microgravity studies may be related to oxygen and micronutrient availability.

Understanding changes in gene expression is an important step in understanding phenotypic changes. The results shown here suggest that swimming speeds and patterns of microbes could be altered under simulated microgravity conditions. Motility and chemotaxis have evolved to provide some microbes an evolutionary advantage. It remains to be seen whether prolonged exposure to simulated microgravity could fundamentally alter both motility gene expression and swimming phenotypes. Experiments involving imaging during and after exposure to simulated microgravity and spaceflight to quantify motility and chemotaxis behaviors remain to be performed.

## References

[1] Rosenzweig JA (2010). Spaceflight and modeled microgravity effects on microbial growth and virulence. Appl. Microbiol. Biotechnol..

[2] Nickerson CA, Ott CM, Wilson JW, Ramamurthy R, Pierson DL (2004). Microbial responses to microgravity and other low-shear environments. Microbiol. Mol. Biol. Rev..

[3] Benoit, M. R. & Klaus, D. M. Microgravity, bacteria, and the influence of motility. *Adv. Space Res.* **39**, 1225-1232 (2007).

[4] Horneck G, Klaus DM, Mancinelli RL (2010). Space microbiology. Microbiol. Mol. Biol. Rev..

[5] Rosenzweig, J. A., Ahmed, S., Eunson, J. & Chopra, K.A. Low-shear force associated with modeled microgravity and spaceflight does not similarly impact the virulence of notable bacterial pathogens. *Appl. Microbiol. Biotechnol.* **98**, 8797–8807 (2014). 10.1007/s00253-014-6025-8 PMC4199916 25149449

[6] Higginson, E. E., Galen, J. E., Levine, M. M. & Tennant, S. M. Microgravity as a biological tool to examine host-pathogen interactions and to guide development of therapeutics and preventatives that target pathogenic bacteria. *Pathog. Dis.* **74**, ftw095 (2016). 10.1093/femspd/ftw095 PMC5985481 27630185

[7] De Angelis, M. et al. Diet influences the functions of the human intestinal microbiome. *Sci. Rep.* **10**, 4247 (2020). 10.1038/s41598-020-61192-y PMC7060259 32144387

[8] Shrivastava A (2018). Cargo transport shapes the spatial organization of a microbial community. Proc. Natl Acad. Sci. USA.

[9] Stecher, B. et al. Flagella and chemotaxis are required for efficient induction of *Salmonella enterica* Serovar Typhimurium Colitis in streptomycin-pretreated mice. *Infect. Immun.* **72**, 4138–4150 (2004). 10.1128/IAI.72.7.4138-4150.2004 PMC427403 15213159

[10] Kao CY (2014). The complex interplay among bacterial motility and virulence factors in different *Escherichia coli* infections. Eur. J. Clin. Microbiol. Infect. Dis..

[11] Duan Q, Zhou M, Zhu L, Zhu G (2013). Flagella and bacterial pathogenicity. J. Basic Microbiol..

[12] Josenhans C, Suerbaum S (2002). The role of motility as a virulence factor in bacteria. Int. J. Med. Microbiol..

[13] Guttenplan SB, Kearns DB (2013). Regulation of flagellar motility during biofilm formation. FEMS Microbiol. Rev..

[14] Akiyama, T. et al. How does spaceflight affect the acquired immune system? *npj Microgravity* **6**, 14 (2020). 10.1038/s41526-020-0104-1 PMC7206142 32411817

[15] Cervantes JL, Hong BY (2016). Dysbiosis and immune dysregulation in outer space. Int. Rev. Immunol..

[16] Voorhies, A. A. et al. Study of the impact of long-duration space missions at the International Space Station on the astronaut microbiome. *Sci. Rep.* **9**, 9911 (2019). 10.1038/s41598-019-46303-8 PMC6616552 31289321

[17] Grimm D (2014). The impact of microgravity-based proteomics research. Expert Rev. Proteom..

[18] Milojevic, T. & Weckwerth, W. Molecular mechanisms of microbial survivability in outer space: a systems biology approach. *Front. Microbiol.* **11**, 923 (2020). 10.3389/fmicb.2020.00923 PMC7242639 32499769

[19] Manzoni C (2018). Genome, transcriptome and proteome: the rise of omics data and their integration in biomedical sciences. Brief Bioinforma..

[20] Raskin DM, Seshadri R, Pukatzki SU, Mekalanos JJ (2006). Bacterial genomics and pathogen. Evolution.

[21] Thein M, Sauer G, Paramasivam N, Grin I, Linke D (2010). Efficient subfractionation of Gram-negative bacteria for proteomics studies. J. Proteome Res..

[22] Pérez-Llarena, F. J. & Bou, G. Proteomics as a tool for studying bacterial virulence and antimicrobial resistance. *Front. Microbiol.* **7**, 410 (2016). 10.3389/fmicb.2016.00410 PMC4814472 27065974

[23] Wilson JW (2002). Microarray analysis identifies *Salmonella* genes belonging to the low-shear modeled microgravity regulon. Proc. Natl Acad. Sci. USA.

[24] Abshire CF (2016). Exposure of *Mycobacterium marinum* to low-shear modeled microgravity: effect on growth, the transcriptome and survival under stress. npj Microgravity.

[25] Rosado, H. et al. Rotating wall vessel exposure alters protein secretion and global gene expression in *Staphylococcus aureus*. *Int. J. Astrobiology* **11**, 71–81 (2012).

[26] Vukanti R, Mintz E, Leff L (2008). Changes in gene expression of *E. coli* under conditions of modeled reduced gravity. Microgravity—Sci. Technol..

[27] Wilson JW (2007). Space flight alters bacterial gene expression and virulence and reveals a role for global regulator Hfq. Proc. Natl Acad. Sci. USA.

[28] Aunins, T. R. et al. Spaceflight modifies *Escherichia coli* gene expression in response to antibiotic exposure and reveals role of oxidative stress response. *Front. Microbiol.* **9**, 310 (2018). 10.3389/fmicb.2018.00310 PMC5865062 29615983

[29] Crabbe A (2011). Transcriptional and proteomic responses of *Pseudomonas aeruginosa* PAO1 to spaceflight conditions involve Hfq regulation and reveal a role for oxygen. Appl. Environ. Microbiol..

[30] Duscher, A. A. et al. Transcriptional profiling of the mutualistic bacterium *Vibrio fischeri* and an hfq mutant under modeled microgravity. *npj Microgravity* **4**, 25 (2018). 10.1038/s41526-018-0060-1 PMC6299092 30588486

[31] Karouia F, Peyvan K, Pohorille A (2017). Toward biotechnology in space: High-throughput instruments for in situ biological research beyond Earth. Biotechnol. Adv..

[32] McKenna A (2010). The Genome Analysis Toolkit: a MapReduce framework for analyzing next-generation DNA sequencing data. Genome Res..

[33] Vaughan, L. K. & Srinivasasainagendra, V. Where in the genome are we? A cautionary tale of database use in genomics research. *Front. Genet.* **4**, 38 (2013). 10.3389/fgene.2013.00038 PMC3604632 23519237

[34] Pinu FR (2019). Systems biology and multi-omics integration: viewpoints from the metabolomics research community. Metabolites.

[35] Ray S (2019). GeneLab: omics database for spaceflight experiments. Bioinformatics.

[36] Zhao K, Liu M, Burgess RR (2007). Adaptation in bacterial flagellar and motility systems: from regulon members to ‘foraging’-like behavior in *E. coli*. Nucleic Acids Res..

[37] Chilcott GS, Hughes KT (2000). Coupling of flagellar gene expression to flagellar assembly in *Salmonella enterica* Serovar Typhimurium and *Escherichia coli*. Microbiol. Mol. Biol. Rev..

[38] Alexandre G, Greer-Phillips S, Zhulin IB (2004). Ecological role of energy taxis in microorganisms. FEMS Microbiol. Rev..

[39] Delong EF (2006). Community genomics among stratified microbial assemblages in the ocean’s interior. Science.

[40] Gilbert JA (2010). The taxonomic and functional diversity of microbes at a temperate coastal site: a ‘multi-omic’ study of seasonal and diel temporal variation. PLoS ONE.

[41] Hammond TG, Hammond JM (2001). Optimized suspension culture: the rotating-wall vessel. Am. J. Physiol. Ren. Physiol..

[42] Guo P, Weinstein AM, Weinbaum S (2000). A hydrodynamic mechanosensory hypothesis for brush border microvilli. Am. J. Physiol.-Ren. Physiol..

[43] Nauman EA (2007). Novel quantitative biosystem for modeling physiological fluid shear stress on cells. Appl. Environ. Microbiol..

[44] Castro SL, Nelman-Gonzalez M, Nickerson CA, Ott CM (2011). Induction of attachment-independent biofilm formation and repression of hfq expression by low-fluid-shear culture of *Staphylococcus aureus*. Appl. Environ. Microbiol..

[45] Allen CA, Niesel DW, Torres AG (2008). The effects of low-shear stress on adherent-invasive *Escherichia coli*. Environ. Microbiol..

[46] Ray P. Schwarz, D. A. W. Rotating bio-reactor cell culture apparatus. United States patent 4988623 (1991).

[47] Herranz R (2013). Ground-based facilities for simulation of microgravity: organism-specific recommendations for their use, and recommended terminology. Astrobiology.

[48] Prewett, T. L., Goodwin, T. J. & Spaulding, G. F. Three-dimensional modeling of T-24 human bladder carcinoma cell line: a new simulated microgravity culture vessel. *J. Tiss. Cult. Meth.* **15**, 29–36 (1993).

[49] Schwarz RP, Goodwin TJ, Wolf DA (1992). Cell culture for three-dimensional modeling in rotating-wall vessels: an application of simulated microgravity. J. Tissue Cult. Methods.

[50] Begley CM, Kleis SJ (2000). The fluid dynamic and shear environment in the NASA/JSC rotating-wall perfused-vessel bioreactor. Biotechnol. Bioeng..

[51] Hoson T, Kamisaka S, Masuda Y, Yamashita M (1992). Changes in plant growth processes under microgravity conditions simulated by a three-dimensional clinostat. Bot. Mag. = Shokubutsu-gaku-zasshi.

[52] van Loon JJWA (2007). Some history and use of the random positioning machine, RPM, in gravity related research. Adv. Space Res..

[53] Barrila J (2010). Organotypic 3D cell culture models: using the rotating wall vessel to study host–pathogen interactions. Nat. Rev. Microbiol..

[54] Barrila, J. et al. Modeling host–pathogen interactions in the context of the microenvironment: three-dimensional cell culture comes of age. *Infect. Immunity* **86**, 00282-18 (2018). 10.1128/IAI.00282-18 PMC6204695 30181350

[55] Grimm D (2014). Growing tissues in real and simulated microgravity: new methods for tissue engineering. Tissue Eng. Part B.

[56] Wolf, D. A. K. S. J. Principles of Analogue and True Microgravity Bioreactors to Tissue Engineering. In C.A. Nickerson, N.R. Pellis, M.C. Ott (Eds.), *Effect of Spaceflight and Spaceflight Analogue Culture on Human and Microbrial Cells*, 39–60 (2016).

[57] Klaus DM (2001). Clinostats and bioreactors. Gravit. Space Biol. Bull..

[58] Wuest SL, Stern P, Casartelli E, Egli M (2017). Fluid dynamics appearing during simulated microgravity using random positioning machines. PLoS ONE.

[59] Ayyaswamy PS, Mukundakrishnan K (2007). Optimal conditions for simulating microgravity employing NASA designed rotating wall vessels. Acta Astronaut..

[60] Barnes, S. J. & Harris, L. P. *Tissue Engineering: Roles*, *Materials, and Applications* (Nova Science Publishers, 2008).

[61] Lynch SV, Mukundakrishnan K, Benoit MR, Ayyaswamy PS, Matin A (2006). *Escherichia coli* biofilms formed under low-shear modeled microgravity in a ground-based system. Appl. Environ. Microbiol..

[62] Gao H, Ayyaswamy P, Ducheyne P (1997). Dynamics of a microcarrier particle in the simulated microgravity environment of a rotating-wall vessel. Microgravity Sci. Technol..

[63] Liu T, Li X, Sun X, Ma X, Cui Z (2004). Analysis on forces and movement of cultivated particles in a rotating wall vessel bioreactor. Biochem. Eng. J..

[64] Borst AG, Van Loon JJWA (2009). Technology and developments for the random positioning machine, RPM. Microgravity Sci. Technol..

[65] Crabbé, A. et al. Response of *Pseudomonas aeruginosa* PAO1 to low shear modelled microgravity involves AlgU regulation. *Environ. Microbiol.* **12**, 1545–1564 (2010). 10.1111/j.1462-2920.2010.02184.x 20236169

[66] Ramirez LES, Lim EA, Coimbra CFM, Kobayashi MH (2003). On the dynamics of a spherical scaffold in rotating bioreactors. Biotechnol. Bioeng..

[67] Friedrich ULD, Joop O, Pütz C, Willich G (1996). The slow rotating centrifuge microscope NIZEMI—a versatile instrument for terrestrial hypergravity and space microgravity research in biology and materials science. J. Biotechnol..

[68] Hemmersbach R, Voormanns R, Häder DP (1996). Graviresponses in Paramecium biaurelia under different accelerations: studies on the ground and in space. J. Exp. Biol..

[69] Pache C (2010). Digital holographic microscopy real-time monitoring of cytoarchitectural alterations during simulated microgravity. J. Biomed. Opt..

[70] Toy, M. F. et al. Dual-mode Digital Holographic and Fluorescence Microscopy for the Study of Morphological Changes in Cells Under Simulated Microgravity. In J.Conchello, C.J. Cogswell, T. Wilson (Eds.), *Three-Dimensional and Multidimensional Microscopy: Image Acquisition and Processing XVII*, **7570** (SPIE, 2010).

[71] Toy MF (2012). Enhanced robustness digital holographic microscopy for demanding environment of space biology. Biomed. Opt. Express.

[72] Yew AG, Atencia J, Hsieh AH (2013). Lab-on-chip clinorotation system for live-cell microscopy under simulated microgravity. Cell. Mol. Bioeng..

[73] Soni A (2014). Conservation of the low-shear modeled microgravity response in Enterobacteriaceae and analysis of the trp genes in this response. Open Microbiol. J..

[74] Wilson, J. W. et al. Media ion composition controls regulatory and virulence response of *Salmonella* in spaceflight. *PLoS One* **3**, e3923 (2008). 10.1371/journal.pone.0003923 PMC2592540 19079590

[75] Grant KC, Khodadad CLM, Foster JS (2014). Role of Hfq in an animal–microbe symbiosis under simulated microgravity conditions. Int. J. Astrobiol..

[76] Tucker DL (2007). Characterization of *Escherichia coli* MG1655 grown in a low-shear modeled microgravity environment. BMC Microbiol..

[77] Tirumalai, M. R. et al. Evaluation of Acquired antibiotic resistance in *Escherichia coli* exposed to long-term low-shear modeled microgravity and background antibiotic exposure. *mBio* **10**, 02637-18 (2019). 10.1128/mBio.02637-18 PMC6336426 30647159

[78] Yim J (2020). Transcriptional profiling of the probiotic *Escherichia coli* Nissle 1917 strain under simulated microgravity. Int. J. Mol. Sci..

[79] Tirumalai MR (2017). The adaptation of *Escherichia coli* cells grown in simulated microgravity for an extended period is both phenotypic and genomic. NPJ Microgravity.

[80] Valentin-Hansen P, Eriksen M, Udesen C (2004). The bacterial Sm-like protein Hfq: a key player in RNA transactions. Mol. Microbiol..

[81] Vogel J, Luisi BF (2011). Hfq and its constellation of RNA. Nat. Rev. Microbiol..

[82] Gottesman S, Storz G (2011). Bacterial small RNA regulators: versatile roles and rapidly evolving variations. Cold Spring Harb. Perspect. Biol..

[83] Chao Y, Vogel J (2010). The role of Hfq in bacterial pathogens. Curr. Opin. Microbiol..

[84] Sittka A, Pfeiffer V, Tedin K, Vogel J (2007). The RNA chaperone Hfq is essential for the virulence of *Salmonella typhimurium*. Mol. Microbiol..

[85] Derosier DJ (1998). The turn of the screw: the bacterial flagellar. Motor.

[86] Girgis HS, Liu Y, Ryu WS, Tavazoie S (2007). A comprehensive genetic characterization of bacterial motility. PLoS Genet..

[87] Rajagopala SV (2007). The protein network of bacterial motility. Mol. Syst. Biol..

[88] Kanehisa M (2006). From genomics to chemical genomics: new developments in KEGG. Nucleic Acids Res..

[89] Soutourina OA, Bertin PN (2003). Regulation cascade of flagellar expression in Gram-negative bacteria. FEMS Microbiol. Rev..

[90] Anderson JK, Smith TG, Hoover TR (2010). Sense and sensibility: flagellum-mediated gene regulation. Trends Microbiol..

[91] Feldman M (1998). Role of flagella in pathogenesis of *Pseudomonas aeruginosa* pulmonary infection. Infect. Immun..

[92] Chaban B, Hughes HV, Beeby M (2015). The flagellum in bacterial pathogens: for motility and a whole lot more. Semin. Cell Dev. Biol..

[93] Ottemann KM, Miller JF (1997). Roles for motility in bacterial–host interactions. Mol. Microbiol..

[94] Pratt LA, Kolter R (1998). Genetic analysis of *Escherichia coli* biofilm formation: roles of flagella, motility, chemotaxis and type I pili. Mol. Microbiol..

[95] Portela, R., Almeida, P. L., Sobral, R. G. & Leal, C. R. Motility and cell shape roles in the rheology of growing bacteria cultures. *Eur. Phys. J. E* **42**, 26 (2019). 10.1140/epje/i2019-11787-9 30810829

[96] Zea, L. et al. Phenotypic changes exhibited by *E. coli* cultured in space. *Front. Microbiol.* **8**, 1598 (2017). 10.3389/fmicb.2017.01598 PMC5581483 28894439

[97] Adler J (1969). Chemoreceptors in bacteria. Science.

[98] Hazelbauer GL (2012). Bacterial chemotaxis: the early years of molecular studies. Annu. Rev. Microbiol..

[99] Wadhams GH, Armitage JP (2004). Making sense of it all: bacterial chemotaxis. Nat. Rev. Mol. Cell Biol..

[100] Wuichet K, Zhulin IB (2010). Origins and diversification of a complex signal transduction system in Prokaryotes. Sci. Signal..

[101] Blount, Z. D. The unexhausted potential of *E. coli*. *eLife* **4**, e05826 (2015). 10.7554/eLife.05826 PMC4373459 25807083

[102] Hayashi, K. et al. Highly accurate genome sequences of *Escherichia coli* K-12 strains MG1655 and W3110. *Mol. Syst. Biol.* **2**, 2006.0007 (2006). 10.1038/msb4100049 PMC1681481 16738553

[103] Grozdanov L (2004). Analysis of the genome structure of the nonpathogenic probiotic *Escherichia coli* strain Nissle 1917. J. Bacteriol..

[104] Sonnenborn U, Schulze J (2009). The non-pathogenic *Escherichia coli* strain Nissle 1917—features of a versatile probiotic. Microb. Ecol. Health Dis..

[105] Tsui H-CT, Leung H-CE, Winkler ME (1994). Characterization of broadly pleiotropic phenotypes caused by an hfq insertion mutation in *Escherichia coli* K-12. Mol. Microbiol..

[106] Yim J (2020). Transcriptional profiling of the probiotic *Escherichia coli* Nissle 1917 strain under simulated microgravity. Int. J. Mol. Sci..

[107] McClelland M (2001). Complete genome sequence of *Salmonella enterica* serovar Typhimurium LT2. Nature.

[108] Nickerson CA (2000). Microgravity as a novel environmental signal affecting *Salmonella enterica* serovar Typhimurium virulence. Infect. Immun..

[109] Sittka A (2008). Deep sequencing analysis of small noncoding RNA and mRNA targets of the global post-transcriptional regulator, Hfq. PLoS Genet..

[110] Monteiro C (2012). Hfq and Hfq-dependent small RNAs are major contributors to multicellular development in *Salmonella enterica* serovar Typhimurium. RNA Biol..

[111] Pacello F (2012). Low-shear modeled microgravity enhances *Salmonella Enterica* resistance to hydrogen peroxide through a mechanism involving KatG and KatN. Open Microbiol. J..

[112] Frye J (2006). Identification of new flagellar genes of *Salmonella enterica* Serovar Typhimurium. J. Bacteriol..

[113] Stover CK (2000). Complete genome sequence of *Pseudomonas aeruginosa* PAO1, an opportunistic pathogen. Nature.

[114] Kim W (2013). Spaceflight promotes biofilm formation by *Pseudomonas aeruginosa*. PLoS ONE.

[115] Hill, I. T., Tallo, T., Dorman, M. J. & Dove, S. L. Loss of RNA chaperone Hfq unveils a toxic pathway in *Pseudomonas aeruginosa*. *J. Bacteriol.* **201**, e00232-19 (2019). 10.1128/JB.00232-19 PMC6755729 31358608

[116] Kato J, Kim H-E, Takiguchi N, Kuroda A, Ohtake H (2008). *Pseudomonas aeruginosa* as a model microorganism for investigation of chemotactic behaviors in ecosystem. J. Biosci. Bioeng..

[117] Francis, V. I., Stevenson, E. C. & Porter, S. L. Two-component systems required for virulence in *Pseudomonas aeruginosa*. *FEMS Microbiol. Lett.* **364**, fnx104 (2017). 10.1093/femsle/fnx104 PMC5812489 28510688

[118] Kim W (2013). Effect of spaceflight on *Pseudomonas aeruginosa* final cell density is modulated by nutrient and oxygen availability. BMC Microbiol..

[119] Ruby, E. G. et al. Complete genome sequence of *Vibrio fischeri*: a symbiotic bacterium with pathogenic congeners. *Proc. Natl. Acad. Sci.* **102**, 3004–3009 (2005). 10.1073/pnas.0409900102 PMC549501 15703294

[120] Foster, J., Wheeler, R. & Pamphile, R. Host–microbe interactions in microgravity: assessment and implications. *Life* **4**, 250–266 (2014). 10.3390/life4020250 PMC4187166 25370197

[121] McFall-Ngai MJ, Ruby EG (1991). Symbiont recognition and subsequent morphogenesis as early events in an animal–bacterial mutualism. Science.

[122] Graf J, Dunlap PV, Ruby EG (1994). Effect of transposon-induced motility mutations on colonization of the host light organ by *Vibrio fischeri*. J. Bacteriol..

[123] Deloney-Marino CR, Visick KL (2012). Role for cheR of *Vibrio fischeri* in the Vibrio–squid symbiosis. Can. J. Microbiol..

[124] Morrison, M. D. & Nicholson, W. L. Meta-analysis of data from spaceflight transcriptome experiments does not support the idea of a common bacterial “spaceflight response”. *Sci. Rep.* **8**, 14403 (2018). 10.1038/s41598-018-32818-z PMC6158273 30258082

[125] Klaus D, Simske S, Todd P, Stodieck L (1997). Investigation of space flight effects on *Escherichia coli* and a proposed model of underlying physical mechanisms. Microbiology.

[126] Zea, L. et al. A molecular genetic basis explaining altered bacterial behavior in space. *PLoS One* **11**, e0164359 (2016). 10.1371/journal.pone.0164359 PMC5091764 27806055

[127] Dijkstra, C. E. et al. Diamagnetic levitation enhances growth of liquid bacterial cultures by increasing oxygen availability. *J. R. Soc. Interface* **8**, 334–344 (2011). 10.1098/rsif.2010.0294 PMC3030818 20667843

[128] Mitchell JG, Kogure K (2006). Bacterial motility: links to the environment and a driving force for microbial physics. FEMS Microbiol. Ecol..

[129] Rossi E, Paroni M, Landini P (2018). Biofilm and motility in response to environmental and host-related signals in Gram negative opportunistic pathogens. J. Appl. Microbiol..

[130] Bains, M., Fernandez, L. & Hancock, R. E. W. Phosphate starvation promotes swarming motility and cytotoxicity of *Pseudomonas aeruginosa*. *Appl. Environ. Microbiol.* **78**, 6762–6768 (2012). 10.1128/AEM.01015-12 PMC3426718 22773629

[131] Marteyn, B., Scorza, F. B., Sansonetti, P. J. & Tang, C. Breathing life into pathogens: the influence of oxygen on bacterial virulence and host responses in the gastrointestinal tract. *Cell. Microbiol.* **13**, 171–176 (2011). 10.1111/j.1462-5822.2010.01549.x 21166974

[132] Lee, C. A. & Falkow, S. The ability of *Salmonella* to enter mammalian cells is affected by bacterial growth state. *Proc. Natl. Acad. Sci.* **87**, 4304–4308 (1990). 10.1073/pnas.87.11.4304 PMC54097 2349239

[133] Deng Y (2016). Complete genome sequence of *Vibrio alginolyticus* ZJ-T. Genome Announc..

[134] Gangaiah, D. et al. *Haemophilus ducreyi* Hfq contributes to virulence gene regulation as cells enter stationary phase. *Mbio* **5**, e01081-01013-e01081 (2014). 10.1128/mBio.01081-13 PMC3950518 24520065

[135] Kim S (2015). hfq plays important roles in virulence and stress adaptation in *Cronobacter sakazakii* ATCC 29544. Infect. Immun..

[136] Wang, C. et al. Hfq, a RNA chaperone, contributes to virulence by regulating plant cell wall–degrading enzyme production, Type VI secretion system expression, bacterial competition, and suppressing host defense response in *Pectobacterium carotovorum*. *Mol. Plant–Microbe Interact.* **31**, 1166–1178 (2018). 10.1094/MPMI-12-17-0303-R 30198820

[137] Osterman IA, Dikhtyar YY, Bogdanov AA, Dontsova OA, Sergiev PV (2015). Regulation of flagellar gene expression in bacteria. Biochemistry.

[138] Caldelari I, Chao Y, Romby P, Vogel J (2013). RNA-mediated regulation in pathogenic bacteria. Cold Spring Harb. Perspect. Med..

[139] Wuest SL, Richard S, Kopp S, Grimm D, Egli M (2015). Simulated microgravity: critical review on the use of random positioning machines for mammalian cell culture. BioMed. Res. Int..

